# Preparing residents for family practice: the role of an integrated “Triple C” curriculum

**Published:** 2013-03-31

**Authors:** Joseph Lee, Colleen McMillan, Loretta M. Hiller, Glenda O’Brien

**Affiliations:** 1 Centre for Family Medicine, Waterloo Region, Ontario, Canada; 2 McMaster University, Hamilton, Ontario, Canada; 3 Wilfrid Laurier University, Waterloo, Ontario, Canada; 4 Aging, Rehabilitation & Geriatric Care Research Centre, Lawson Health Research Institute, London, Ontario, Canada

## Abstract

**Background:**

There is limited understanding of the impact of Triple C competency-based curriculums on the preparation of residents for family practice. This paper describes a competency-based curriculum within an integrated longitudinal block design and presents preliminary evaluation data on the impact of this curriculum on preparedness for family practice.

**Methods:**

First and second year family medicine residents were surveyed as a component of a year-end program evaluation to assess the extent to which the residency program is preparing them to engage in a variety of practice domains, the likelihood that they would engage in these domains, and the extent to which this residency program is comprehensive, relevant to their development as a family physician, and promotes interprofessional practice.

**Results:**

Residents perceived themselves as prepared to engage in most practice areas and their intentions to engage in various practice domains were positively correlated to their ratings of preparedness. Ratings reflected that residents perceived this program as comprehensive and relevant to their development as a family physician and they perceived a high degree of encouragement for interprofessional practice.

**Conclusions:**

This study provides some preliminary evidence that an integrated competency-based curriculum, with an emphasis on interprofessional practice has the potential to effectively prepare residents for practice in family medicine.

## Introduction

The College of Family Physicians of Canada Working Group on Postgraduate Curriculum Review (WGCR) has recommended that all residency training programs establish Triple C competency-based curriculums, which are comprehensive, focused on continuity of education and patient care, and centred in family medicine,^1^–^3^ to ensure that family medicine residents are optimally prepared to provide comprehensive care through the achievement of a full range of clinical competencies and to facilitate social responsibility.^4^ Training experiences emphasize continuity, in terms of perceiving learning as something that continues overtime in practice as well as in terms of continuity of patient care. Centering education in family medicine by using family physician educators within a family medicine setting, augmented with teaching outside of family medicine and with other disciplines, further facilitates the achievement of clinical competencies. There is currently some debate about how to define competency; however, it is generally agreed that competency has multiple components including knowledge, skills, reflection in daily practice, and appropriate values in decision-making.^5^ Although a number of methods for assessing competency exist, clinical evaluation in competency-based curriculums is continually evolving.^6^

The Kitchener-Waterloo Centre for Family Medicine, part of the multi-site McMaster University family medicine program, introduced the Triple C curriculum in 2007 and offers clinical experiences across practice areas that are enhanced by horizontal experiences in the core family medicine block and a new integrated family medicine block. The Triple C curriculum within the CFFM emphasizes inter-professional education and teamwork facilitated by the use of family medicine-centred, inter-professional clinical care that incorporates care of the elderly, chronic disease prevention and management, maternal and child health, and mental health programs; clinical teaching is provided to family medicine residents as well as residents from other disciplines. The College of Family Physicians of Canada Working Group on Postgraduate Curriculum Review R supports interprofessional practice as a mechanism by which to meet the health care needs of Canadians.^3^ Family medicine education within the CFFM conforms to the Triple C curriculum, and was designed to meet the criteria for testing and competence in family medicine.^7^ Competency has been assessed by “field notes” whereby preceptors assess residents on the various domains of family medicine. Evaluations have been conducted every 6 months using a “resident portfolio” which includes In Training Evaluation Reports (ITER’s), guided resident reflections, a review of educational objectives, and a checklist of required competencies such as leadership activities, quality assurance, palliative care cases, family medicine obstetrics cases, review of all field notes, and a procedural log.

Learning in integrated, or longitudinal, blocks of time allows experience in other disciplines to be readily integrated into the family medicine context and occurs for the most part in the setting in which it will be ultimately applied. In contrast, in ‘block” or “rotational” models of training 8,9 residents learn from specialists in sequential, discipline specific blocks of time what they need to know in application to the family medicine context. Although it is not known whether integrated experiences are a more effective learning method than block rotations, there is some evidence that integrated learning experiences enhance learner-patient relationships and patient-centered care,^10^ provide enhanced opportunities to develop context-specific clinical reasoning and cross-disciplinary competencies,^11^ and facilitate clinical skill assessment.^12^

Although the CFFM residency program is primarily integrated, some components are not. For example, general pediatrics, obstetrics/gynecology, and hospitalist medicine were judged to be better learning experiences when conducted in a block rotation with daily patient continuity and consolidated learning. A typical week in this family medicine program is presented in Table 1. In Year 1 of the program, general pediatrics, emergency medicine, and obstetrics/gynecology blocks have emphasized care in a family medicine-specific context. In rotations such as pediatrics and obstetrics, where preceptors are not family physicians, the program has conducted faculty development with these preceptors to introduce the concept of teaching within the context of family medicine. The family medicine block has been increased from four months to six months and includes horizontal experiences in other areas of clinical care. The two month internal medicine block has been revised as a hospitalist medicine block taught principally by family physicians involved in typical family medicine hospital work. In Year 2, the family medicine time has been increased from six months to eight months and the remaining four months consists of family medicine-centred selectives and electives. The program provides exposure to comprehensiveness in various practice settings such as medical offices, hospitals, emergency rooms, house calls, long-term care facilities and through “cradle to grave” experiences in various settings. Residents conduct continuity clinics within family medicine for the entire 2 year program for a minimum of ½ day per week.

Given the paucity of studies that explore the impact of the new triple C curriculum and competency-based residency curriculums, a preliminary evaluation was undertaken to assess residents’ perceptions of this curriculum and its impact on preparedness for family practice.

## Methods

A survey methodology was employed in this study. Questions evaluating the competency-based model were added to an anonymous standardized program evaluation form, which all residents in year 1 and 2 completed at the end of the 2010–2011 academic year. Residents were asked to rate (7-point scale: 1 = not at all, 7 = extremely/completely) the comprehensiveness of the program, in terms of integrating multiple practice domains and settings, the relevance of the rotations/practice experiences, the extent to which this residency program prepares them for these domains and the likelihood that they would engage in them, as well as activities in their family medicine practice and the extent to which this residency program encouraged them to engage in interprofessional practice. All questions were rated on a 7-point scale (1 = not at all, 7 = extremely/completely). Residents were also asked to identify how they intended to practice upon graduation (response choices: Group Practice – FHT, FHO, CHC etc; Walk-in clinic; Hospital; Locums; Solo Practice; Other).

## Results

**Figure 1:**
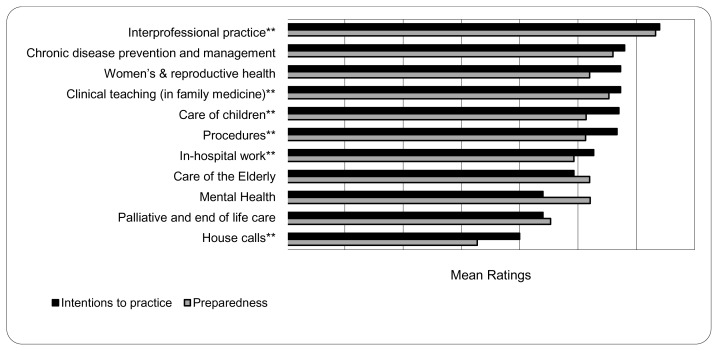
Ratings* of preparedness for and intentions to engage in various practice domains and activities (*n* = 15) * As rated on a 7-point scale: 1 = not at all, 7 = extremely well prepared. **Positive correlations: Interprofessional practice, *r* = 0.62, *p* < 0.01; Clinical teaching, *r* = 0.77, *p* < 0.001; Care of children, *r* = 0.56, *p* < 0.05; Procedures, *r* = 0.60, *p* < 0.05; In-hospital work, *r* = 0.68, *p* < 0.01; House calls, *r* = 0.54, *p* < 0.05.

Out of a total of 19 residents, 8 first-year residents and 7 second-year residents completed the questionnaire (71% response rate). Figure 1 presents residents’ mean ratings of their preparedness for, and intentions to engage in, various practice domains and activities. Mean ratings of their preparedness to engage in various practice domains and activities ranged from 3.27 (*SD* = 1.5) for House Calls to 6.33 (*SD* = 0.72) for Interprofessional Practice. Mean ratings of residents’ intentions to engage in the various practice domains and activities ranged from 4.00 (*SD* = 1.6) for House Calls to 6.40 (*SD* = 0.72) for Interprofessional Practice. There were no statistically significant differences in these ratings between first and second-year residents. Intentions to practice were positively correlated with ratings of preparedness for a number of practice areas (see Figure 1).

**Figure 2:**
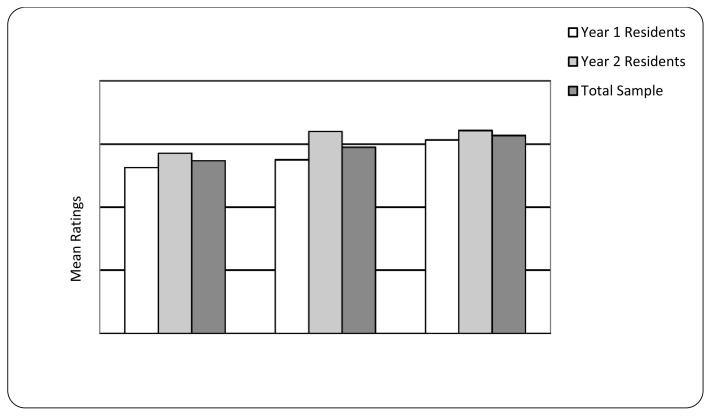
Ratings* of program comprehensiveness, relevance and encouragement to engage in interprofessional practice (*n* = 15) *As rated on a 7-point scale: 1 = not at all, 7 = extremely comprehensive/relevant/completely.

Residents’ perceived the program as moderately comprehensive (*M* = 5.5, *SD* = 0.74), highly relevant to their development as a family physician (*M* = 5.9, *SD* = 0.74) and perceived a high degree of encouragement for interprofessional practice (*M* = 6.27, *SD* = 0.70; see Figure 2). Although there were no statistically significant differences between first and second-year residents in ratings of comprehensiveness of the program or encouragement for interprofessional practice, Year-2 residents had significantly higher mean ratings of the relevance of the program to their development as a family physician (*M* = 6.4, *SD* = 0.53, *n* = 7) than Year-1 residents (*M* = 5.5, *SD* = 0.53, *n* = 8), *F*(1,13) = 11.27, *p* < 0.01.

All residents indicated that upon graduation they intended to practice within a group setting such as a family health team, family health organization or community health centre. Four residents (2 first-year and 2 second-year) also indicated intentions to work in a hospital and as locums.

## Discussion

Acknowledging that the small size limits generalization from this study, these preliminary findings nonetheless describe a potential relationship between learning in an interprofessional environment and the desire to work in such an environment upon graduation. Our findings highlight the importance of exploring this relationship further, particularly with family physicians who have completed this residency program and have established their clinical practice, using more rigorous research designs, such as the use of randomized controlled and quasi-experimental designs with larger sample sizes to compare the outcomes of different types of curriculums and clerkships, potentially using those programs that have not adopted a Triple C competency-based curriculum as a comparison group. A significant limitation to this study is the focus on self-report rather than actual outcomes related to practice domains and activities explored in this study. Qualitative studies providing a more in-depth analysis of impacts and longitudinal studies that survey residents once in practice can elucidate further the extent to which a Triple C curriculum prepares residents for the realities of family medicine.

Survey questions purposefully did not explore individual rotations or experiences but looked at the broader categories of practice that the residents would experience across multiple areas during their two-year program. Key among the findings were high perceptions of preparedness and intention to practice in the areas of Interprofessional Practice, Chronic Disease Prevention & Management, Women’s and Reproductive Health, and Clinical Teaching, with lower ratings of preparedness and intentions to practice Palliative Care and to conduct House Calls; these findings have led to new initiatives such as the inter-professional supportive care clinic. Given residents’ very high intentions to work in a group practice that collaborated with other health care disciplines, this program has been successful in promoting interprofessional practice. Overall, both first and second-year residents ranked the Kitchener-Waterloo site curriculum highly as being relevant and comprehensive to their future careers as family physicians.

This study found higher levels of preparedness for family medicine practice than earlier studies examining this prior to the introduction of Triple C curriculum.^13^;^14^ Although there could potentially be a number of reasons for this, including a greater emphasis more time in family medicine block rotations and greater emphasis on family medicine experiences, as well as more support for and exposure to interprofessional care teams, the potential role of the Triple C curriculum in enhancing self-perceptions of preparedness should not be minimized. Although residents in this study were most likely to express intentions to engage in practice domains for which they felt well prepared, it is difficult to identify the causal relationship here. It may be the case that residents put less effort into preparations for practice domains in which they have low intentions of engaging.

This exploration of potential impacts of a Triple C curriculum suggests that an integrated competency-based curriculum, with an emphasis on interprofessional practice can effectively prepare residents for practice in family medicine.

## Figures and Tables

**Table 1:** A typical week in the family medicine program (Year 1 & Year 2)

	Monday	Tuesday	Wednesday	Thursday	Friday
**Morning** Year 1	Gynecology Clinic	Family Medicine Clinic	Family Medicine Clinic	Normal Newborn Clinic	Pediatric Psychiatry
Year 2	Geriatrics Clinic	Long-Term Care	Family Medicine Clinic	Women’s Health Clinic	
**Noon** Year 1& 2			Resident Rounds		
**Afternoon** Year 1	Family Medicine Clinic	Maternal Child Clinic	Behavioral Science	Family Medicine Clinic	Family Medicine Clinic
Year 2	Family Medicine Clinic	Family Medicine Clinic	Behavioral Science	Family Medicine Clinic	Memory Clinic
**Evening** Year 1		Family Medicine Obstetrics Call			
Year 2				On-Call	
